# Efficacy and safety of intensity Modulated Radiation therapy combined with Concurrent Chemoradiotherapy in the treatment of Recurrent Cervical Cancer

**DOI:** 10.12669/pjms.39.4.6784

**Published:** 2023

**Authors:** Ge Jin, Kuixiu Li, Shuhuai Niu, Xiaomei Fan, Yunfeng Guo

**Affiliations:** 1 Ge Jin, Department of Gynecology and Oncology, The Fourth Hospital of Hebei Medical University, Shijiazhuang, 050000, Hebei, P.R. China; 2 Kuixiu Li, Department of Gynecology and Oncology, The Fourth Hospital of Hebei Medical University, Shijiazhuang, 050000, Hebei, P.R. China; 3 Shuhuai Niu, Department of Gynecology and Oncology, The Fourth Hospital of Hebei Medical University, Shijiazhuang, 050000, Hebei, P.R. China; 4 Xiaomei Fan, Department of Gynecology and Oncology, The Fourth Hospital of Hebei Medical University, Shijiazhuang, 050000, Hebei, P.R. China; 5 Yunfeng Guo, Department of Gynecology and Oncology, The Fourth Hospital of Hebei Medical University, Shijiazhuang, 050000, Hebei, P.R. China

**Keywords:** Intensity Modulated Radiation Therapy, Concurrent Chemoradiotherapy, Recurrent Cervical Cancer

## Abstract

**Objective:**

To evaluate the clinical value of intensity modulated radiation therapy (IMRT) combined with concurrent chemoradiotherapy in the treatment of recurrent cervical cancer.

**Methods:**

This was a retrospective study. Eighty patients with recurrent cervical cancer were recruited and randomly divided into two groups: the experimental group and the control group, with 40 cases in each group at The Fourth Hospital of Hebei Medical University from April, 2017 to April, 2022. Patients in the control group were only given IMRT, while those in the experimental group were given concurrent chemoradiotherapy with paclitaxel and cisplatin based on IMRT. All patients were evaluated for clinical efficacy, adverse drug reactions, and differences in the levels of SCC-Ag, CEA and CA724 and other tumor markers before and after treatment.

**Results:**

The total effective rate in the experimental group was significantly better than in the control group (p=0.02). The incidence of adverse reactions was 40% in the experimental group and 32.5% in the control group, with no statistically significant difference (p=0.48). After treatment, the levels of tumor markers in the experimental group were significantly lower than those in the control group, with a statistically significant difference (p=0.00). The three years survival rate was 80% in the experimental group and 55% in the control group (p=0.03). The five years survival rate was 65% in the experimental group and 42.5% in the control group, with a statistically significant difference (p=0.04).

**Conclusion:**

Intensity modulated radiation therapy (IMRT) combined with concurrent chemoradiotherapy is a safe and effective regimen for recurrent cervical cancer, boasting significant clinical efficacy, reduced tumor markers, no significant increase in adverse reactions, and significantly improved three-years and five years survival rate.

## INTRODUCTION

Cervical cancer, a malignant tumor occurring in the cervical canal, is a common gynecological disease, accounting for more than 50% of malignant tumors of the reproductive system.^1^ It has been shown in relevant studies^2^ that cervical cancer is the third most common female malignant tumor in the world, and its fatality rate ranks first among female malignant tumors. Most cases of cervical cancer can be prevented with human papillomavirus (HPV) vaccination, routine screening and treatment of precancerous lesions. Nevertheless, cervical cancer is prevalent in developing countries and has a high recurrence rate due to inadequate screening programmes in many underdeveloped regions of the world. As a result, the clinical diagnosis and treatment of cervical cancer in such areas is exceedingly troublesome.^3^ Currently, radiotherapy is clinically preferred for the treatment of recurrent cervical cancer.^4^

However, Marshall et al.^5^ argued that radiotherapy alone was not ideal in a comprehensive way. Despite improved disease control rate under the premise of increasing radiation dose, serious adverse effects can also be caused to surrounding tissues and organs. Some patients are still in serious condition after radiotherapy, with a still low five years survival rate.^6^ In view of this, radiotherapy combined with other treatments has been highlighted in clinical studies on its effect of improving the clinical efficacy of recurrent cervical cancer and prolonging the survival period of patients.^7^ With the continuous advancement of medical technology, concurrent chemoradiotherapy has been gradually applied in the treatment of cervical cancer by virtue of its advantages in significantly improving the cure rate.

In this study, based on previous studies, the efficacy and safety of IMRT combined with concurrent chemoradiotherapy in the treatment of recurrent cervical cancer were observed, and its effect on serum tumor marker levels in patients with recurrent cervical cancer was probed into.

## METHODS

This was a retrospective study. Eighty patients with recurrent cervical cancer admitted to The Fourth Hospital of Hebei Medical University from April, 2017 to April, 2022 were recruited and randomly divided into two groups: the experimental group and the control group, with 40 cases in each group. Patient data including demographic data, diagnosis of primary cancer and baseline dosage were retrieved from electronic medical record systems. Patients in the experimental group were aged from 45 to 75 years, with an average age of 60.46±10.82 years, while those in the control group were aged from 43 to 72 years, with an average age of 58.74±10.47 years. No significant difference was observed in the comparison of general data between the two groups, which was comparable (Table-I). The study was approved by the Institutional Ethics Committee of The Fourth Hospital of Hebei Medical University (No.: 2018MZC147: Date: November 28, 2018), and written informed consent was obtained from all participants.

**Table-I:** Comparative analysis of the general data between the two groups (*χ̅*±*S*) n=40.

Indicators	Experimental group	Control group	t/χ^2^	p
Age (years old)	60.46±10.82	58.74±10.47	0.72	0.47
***Pathological type***				
Squamous cell carcinoma	23 (57.5%)	21 (52.5%)	0.20	0.65
Adenocarcinoma	12 (30%)	13 (32.5%)	0.06	0.81
Other types	5 (12.5%)	4 (10%)	0.13	0.72
***Differentiation grade***				
Well differentiated	26 (65%)	23 (57.5%)	0.47	0.49
Moderately differentiated	14 (35%)	17 (42.5%)		
***Clinical stage***				
II	12 (30%)	14 (35%)	0.23	0.63
III	16 (40%)	13 (32.5%)	0.49	0.46
IV	12 (30%)	13 (32.5%)	0.06	0.81

p>0.05.

### Inclusion criteria:

- Patients meeting the diagnostic criteria for cervical cancer.^8^
- Patients with recurrent cervical cancer confirmed by pathological or cytological
- examination and clinical stage II-IV.
- Patients with clear lesions detected by imaging examination (CT or MRI) and
- whose size can be accurately assessed.^9^
- Patients with good physical condition and self-care ability (KPS score≥70) and an
- expected survival time of more than six months.
- Patients with complete case data and follow-up data.
- Patients aged<75 years old.

### Exclusion criteria:

- Patients with a poor constitution and unstable vital signs who cannot tolerate
- treatment.
- Patients with malignant tumors in other systems.
- Patients with severe underlying diseases.
- Patients with mental or nervous system abnormalities or who are unable to
- complete the study due to other reasons.
- Patients who have recently taken drugs that affect the results of the study, such as
- hormones and immunosuppressants.
- Patients during pregnancy or lactation.

Patients in the experimental group were given IMRT combined with concurrent chemoradiotherapy, and the specific plan was as follows: 1. IMRT: The patient was supine on the body membrane rack, with hands crossed on the chest, and the pelvic cavity was fixed with a body membrane. The positioning laser was directed at the selected reference point on the body surface of the body film to place markers, and enhanced scanning was performed with a scanning layer thickness of 3mm. After scanning, the images were sent to the TPS system. A plan was designed to outline the important organs such as the small intestine, rectum and bladder. The target area (GTV) was delineated layer by layer by radiotherapy doctors.^10^

On the basis of GTV, 0.5-1.0 cm was externally placed to form a planned target area (PTV), protecting tissues including the rectum, colon, small intestine, bladder, bilateral femoral head, spinal cord, both kidneys, stomach, liver, lungs and heart. A 95% dose line was used to wrap the PTV by applying the German Siemens ONCON linear accelerator (6MV, X-ray). Five to seven coplanar irradiation fields were set up, and the tumor dose was 45-50 Gy, 1.8 or 2 Gy/time, once a day, five times a week. Concurrent chemoradiotherapy regimen: On day one, intravenous infusion of paclitaxel 70mg/m2, intravenous infusion of cisplatin 70 mg/m^2^, every 21 days as a cycle, with a total of two cycles.

Patients in the control group received IMRT alone, with five to seven coplanar irradiation fields. The tumor dose was 45-50 Gy, 1.8 or 2 Gy/time, once a day, five times a week. Routine blood tests and examinations of liver and kidney function, electrocardiogram, and chest X-ray were performed between the two groups before and after treatment.

### Observation indicators:

1) Evaluation of clinical efficacy: All patients underwent abdominal and pelvic CT or MRI monthly after treatment to compare the changes in tumor size. Tumors were evaluated according to Response Evaluation Criteria Solid Tumors 1.0 (RECIST1.0):^11^ Complete response (CR): complete disappearance of lesions; Partial response (PR): 30% decrease in the sum of the measured diameters of target lesions relative to baseline; Stable disease (SD): 25%-50% reduction in the maximum diameter of the lesions; Progression disease (PD): at least 20% increase in the sum diameters of all target lesions, and an increase of more than 5mm in the absolute value of the sum diameters (or the appearance of new lesions). Total effective rate = (CR+PR) cases/total cases × 100%. 2): Evaluation of adverse drug reactions: Adverse drug reactions of the two groups within one month after medication were recorded, including anemia, fever, WBC reduction, gastrointestinal symptoms, liver and kidney dysfunction. etc.; 3) Comparative analysis of tumor marker levels: Fasting blood was taken in the morning before and after treatment to detect the levels of SCC-AG, CEA and CA724, and the differences between the two groups before and after treatment were compared and analyzed. 4) Follow-up: All patients were followed up for five years until death or follow-up endpoint, and the three years and five years survival rates were compared between the two groups.

### Statistical analysis:

All data in this study were analyzed with SPSS 20.0 software, and measurement data were expressed as (*χ̅*±*S*). Two independent samples t-test was employed for inter-group data analysis, paired t-test was utilized for intra-group data analysis, while χ^2^ was used for rate comparison. P<0.05 indicates a statistically significant difference.

## RESULTS

The comparative analysis of the efficacy of the two groups was shown in Table-II, indicating that the total effective rate in the experimental group was 72.5% after treatment, which was significantly better than 47.5% in the control group, with a statistically significant difference (p=0.02).

**Table-II:** Comparative analysis of the efficacy of the two groups (*χ̅*±*S*) n=40.

Group	CR	PR	SD	PD	Total effective rate*
Experimental group	10	19	8	3	29 (72.5%)
Control group	9	10	13	8	19 (47.5%)
*χ* ^2^					5.21
*p*					0.02

* p<0.05.

The incidence of adverse drug reactions between the two groups after treatment was compared and analyzed, indicating that the incidence of adverse reactions was 40% in the experimental group and 32.5% in the control group, with no statistically significant difference (p=0.48) (Table-III).

**Table-III:** Comparative analysis of adverse drug reactions between the two groups (*χ̅*±*S*) n=40.

Group	Anemia	Liver and kidney insufficiency	Fever	WBC reduction	Gastrointestinal reaction	Incidence*
Experimental group	4	2	0	6	4	16 (40%)
Control group	3	2	1	5	2	13 (32.5%)
*χ* ^2^						0.50
*p*						0.48

* p>0.05

No significant difference was observed in the levels of SCC-AG, CEA and CA724 between the two groups before treatment (p>0.05). After treatment, the above indicators in the experimental group decreased significantly compared with the control group, with a statistically significant difference (p=0.00) (Table-IV).

**Table-IV:** Comparative analysis of tumor marker levels between the two groups before and after treatment (*χ̅*±*S*) n=40.

Indicators	Time	Experimental group	Control group	t	p
SCC-Ag (ng/ml)	Before treatment	6.48±1.73	6.79±1.41	0.88	0.37
	After treatment*	1.46±0.37	4.28±1.26	13.58	0.00
CEA (ug/L)	Before treatment	8.78±1.62	8.65±1.37	0.39	0.70
	After treatment*	2.20±0.64	4.74±0.66	17.47	0.00
CA724 (U/ml)	Before treatment	26.45±4.37	26.31±4.75	0.14	0.89
	After treatment*	7.31±2.18	14.03±5.63	0.74	0.00

* p<0.05.

The follow-up results of the two groups showed that the three years survival rate was 80% in the experimental group and 55% in the control group (p=0.03). The five years survival rate was 65% in the experimental group and 42.5% in the control group, with a statistically significant difference (p=0.04) (Table-V).

**Table-V:** Comparative analysis of the three years and five years survival rates of the two groups (*χ̅*±*S*) n=40.

Group	3-years survival rate*	5-years survival rate*
Experimental group	32(80%)	26(65%)
Control group	22(55%)	17(42.5%)
*χ* ^2^	4.71	4.07
*p*	0.03	0.04

* p<0.05.

## DISCUSSION

It was confirmed in our study that the total effective rate of IMRT combined with concurrent chemoradiotherapy group (experimental group) was 72.5%, which was significantly better than 47.5% of the IMRT group (control group) alone (p=0.02). The incidence of adverse reactions was 40% in the experimental group and 32.5% in the control group, with no statistically significant difference (p=0.48). After treatment, the levels of tumor markers such as SCC-AG, CEA and CA724 in the experimental group were significantly lower than those in the control group, with a statistically significant difference (p=0.00). The three years survival rate was 80% in the experimental group and 55% in the control group (p=0.03). The five years survival rate was 65% in the experimental group and 42.5% in the control group, with a statistically significant difference (p=0.04).

Cervical cancer is a common malignant tumor in gynecology clinic, which frequently makes inroads in middle-aged women. According to relevant surveys and studies, cervical cancer in recent years has gradually presented a trend of increasing incidence and invasion of young groups.^12^ When it comes to the occurrence of cervical cancer, it has a close bearing on sexual behavior, number of deliveries, and HPV virus infection.^13^ Clinically, surgery and radiotherapy are the preferred treatment methods for cervical cancer. Under normal circumstances, surgery is mostly used for patients with early-stage cervical cancer, boasting ideal efficacy, low recurrence rate and prolonged survival.^14^ Nevertheless, early-stage cervical cancer is often asymptomatic with a smooth cervix that is indistinguishable from cervical columnar ectopy. Patients with cervical cast carcinoma are easily missed or misdiagnosed due to normal cervical appearance.^15^ With the development of lesions, patients with intermediate and advanced cervical cancer are prone to relapse after treatment.

Plenty of new radiotherapy methods have emerged in recent years to improve the clinical control rate of recurrent cervical cancer and reduce the adverse effects of radiotherapy. Intensity modulated radiation therapy (IMRT) is widely used in the radiotherapy of various tumors with its principle of fine division of the radiotherapy area. In the course of radiotherapy, different radiation doses for corresponding target areas may protect normal tissues and increase the local dose of tumors.^16^ A study by Liu et al.^17^ concluded that the IMRT technique boasted of shortening the total treatment time and improving tumor control rate and survival rate without increasing the radiotoxicity of normal tissues. Adverse events frequently associated with this technique included nausea, vomiting, alopecia, neutropenia, and leukopenia, most of which were grade one or two in intensity, with few serious adverse events.

Radiotherapy is an essential measure in the treatment of various stages of cervical cancer. However, it is not ideal for locally advanced or intermediate advanced patients, which can be attributed to the inability of radiotherapy to kill local or subclinical metastatic lesions outside the irradiation field. These lesions are also the underlying cause of tumor metastasis or recurrence.^18^ It has been pointed out in relevant literature that for recurrent cervical cancer, the increased radiation dose is often needed to improve the efficacy due to the large tumor size, the insufficient internal blood supply of tumor cells, hypoxia and low sensitivity to radiation.^19^ However, the increased dose may give rise to a significant increase in adverse reactions, leading to intolerance by patients.^20^ For this reason, chemotherapy is not ideal for recurrent cervical cancer.

Concurrent chemoradiotherapy is widely sought after due to its characteristics such as controlling distant metastasis of lesions, killing tumor cells outside the irradiated area, and controlling the repair and proliferation of tumor cells after radiotherapy. Meanwhile, chemotherapeutic drugs can effectively reduce the proportion of hypoxic cells, shrink the size of the tumor and enhance the sensitivity of tumor cells to radiotherapy rays via cytotoxicity. Furthermore, radiotherapy boasts of promoting the synchronization of tumor cell sensitivity cycle with radiotherapy, lowering radiotherapy dose and reducing the resulting adverse reactions.^21^ Therefore, concurrent chemoradiotherapy is an effective treatment regimen for cervical cancer that can avoid cross-tolerance.

According to the study of Rosen et al.^22^, patients with advanced and recurrent cervical cancer are at a poor prognosis. In this regard, radiotherapy combined with concurrent chemotherapy with paclitaxel and cisplatin can be utilized to prolong the survival of patients with persistent, recurrent or metastatic disease. The study by Yamamoto et al.^23^ confirmed that radiotherapy combined with platinum-based chemotherapy had no evident increase in side effects compared with radiotherapy alone. It was believed by Lazzari et al.^24^ that in clinical practice, IMRT combined with concurrent chemoradiotherapy showed lower toxicity without reducing efficacy in patients with recurrent cervical cancer after surgery. SCC-Ag, CEA and CA72-4 are the main metabolites of tumor cells, and their levels represent tumor volume development. According to related studies^25^, the levels of SCC-Ag, CEA and CA72-4 were significantly higher in patients with cervical cancer than in normal people. These indicators can be used as disease detection-specific indicators, and their reduction suggests a favorable prognosis.^26^

### Limitations of the study:

It includes a small sample size and with the deepening of people’s understanding of the disease, some new therapeutic methods such as immunotherapy have not been included in the study for some reasons. In future large sample sizes will be included in the study, and related research content will be further increased in order to ameliorate the quality of life and survival benefits of such patients.

## CONCLUSIONS

Intensity modulated radiation therapy (IMRT) combined with concurrent chemoradiotherapy is a safe and effective regimen for recurrent cervical cancer, boasting significant clinical efficacy, reduced tumor markers, no significant increase in adverse reactions, and significantly improved three years and five years survival rate.

### Authors’ Contributions:

**GJ and YG:** Designed this study, prepared this manuscript, are responsible and accountable for the accuracy and integrity of the work.

**KL and SN** collected and analyzed clinical data.

**XF** participated in acquisition, analysis, interpretation of data and draft the manuscript.
